# Long-Term Survival by Number of Immune Checkpoint Inhibitors in PD-L1–Negative Metastatic NSCLC: A Systematic Review and Meta-Analysis

**DOI:** 10.1001/jamanetworkopen.2024.57357

**Published:** 2025-02-12

**Authors:** Ben Ponvilawan, Dhruv Bansal, Nagla Abdel Karim, Rahul Ramakrishnan, Janakiraman Subramanian

**Affiliations:** 1 Department of Internal Medicine, University of Missouri–Kansas City School of Medicine, Kansas City; 2 St Luke’s Cancer Institute, University of Missouri–Kansas City School of Medicine, Kansas City; 3 Inova Schar Cancer Institute, Fairfax, Virginia; 4 Georgia Institute of Technology, Atlanta

## Abstract

This systematic review and meta-analysis evaluates long-term survival outcomes by number of immune checkpoint inhibitors among patients with programmed death-ligand 1 (PD-L1)–negative non–small cell lung cancer (NSCLC).

## Introduction

Immune checkpoint inhibitors (ICI) with or without chemotherapy is the current standard of care first-line treatment for patients with metastatic non–small cell lung cancer (NSCLC) without actionable variants. However, patients with negative programmed death-ligand 1 (PD-L1) status are known to experience worse survival outcomes compared with those with positive PD-L1 status despite the addition of ICI to chemotherapy.

With multiple front-line ICI treatment regimens available, the optimal treatment regimen for PD-L1–negative patients has yet to be defined. It is also unclear whether cytotoxic T-lymphocyte–associated protein inhibition, in addition to targeting PD-1/PD-L1 axis, could yield further survival benefits. With the availability of long-term survival data from several phase 3 studies evaluating ICI plus chemotherapy in patients with advanced NSCLC, we studied whether there was a difference between the different regimens in treating patients with PD-L1–negative NSCLC.

## Methods

This systematic review and meta-analysis followed the Preferred Reporting Items for Systematic Reviews and Meta-analyses (PRISMA) reporting guideline, with the study review and selection process described in eFigure 1 in Supplement 1. We included phase 3 randomized clinical trials (RCTs) from 2022 to 2024 that reported 5-year overall survival (OS) or progression-free survival (PFS) of patients with advanced or metastatic PD-L1–negative NSCLC who received an ICI-containing regimen compared with chemotherapy alone. Patients with indeterminate PD-L1 status were excluded from the analysis. Pooled hazard ratios (HR) for OS and PFS and 95% CIs were calculated using the generic inverse variance method. Subgroup analysis based on histological subtype for OS and PFS was further performed if at least 3 eligible studies were found. Cochran *Q* test was used to determine statistical heterogeneity, with 2-sided ; values less than .05 indicating statistical significance for heterogeneity. R version 4.3.2 (R Project for Statistical Computing) and meta package version 7.0-0 were used for analysis.

## Results

Six RCTs consisting of 1684 patients met the criteria and were included in the meta-analysis.^1,2,3,4,5,6^ A total of 1480 and 1228 events were observed for OS and PFS, respectively. The study characteristics, along with the study quality assessment, are summarized in the Table. Patients who received ICIs exhibited superior OS and PFS compared with those who did not (pooled HR, 0.75; 95% CI, 0.66-0.85; *I*^2^ = 41% and HR, 0.72; 95% CI, 0.64-0.81; *I*^2^ = 0%, respectively). Doublet ICI had a lower HR for OS than single-agent ICI, but the results were not statistically significant (pooled HR, 0.69; 95% CI, 0.60-0.79 vs HR, 0.80; 95% CI, 0.66-0.95; *P* for subgroup differences = .21) (Figure, A and B). Interestingly, among studies evaluating single-agent ICI plus chemotherapy, KEYNOTE 189 had better OS compared with other studies (HR 0.55; 95% CI, 0.39-0.77). Leave-one-out analysis excluding KEYNOTE-189 resulted in reduced OS benefits (pooled HR 0.86; 95% CI, 0.75-0.99; *I*^2^ = 0%).

**Table.** Summary of Characteristics of the Studies Included in the Meta-Analysis

Characteristic	POSEIDON	CheckMate 9LA	CheckMate227	KEYNOTE-189	KEYNOTE-407	CameL
Source	Peters et al,^1^ 2023	Reck et al,^2^ 2024	Brahmer et al,^3^ 2022	Garassino et al,^4^ 2023	Novello et al,^5^ 2022	Zhou et al,^6^ 2024
NCT	NCT03164616	NCT03215706	NCT02477826	NCT02578680	NCT02775435	NCT03134872
No. of patients	DUR, TRE, and CMT: 125; DUR and CMT: 113; CMT: 130	NIV, IPI, and CMT: 135; CMT: 129	NIV and IPI: 187; NIV and CMT: 177; CMT: 186	PEM and CMT: 127; CMT: 63	PEM and CMT: 95; CMT: 99	CAM and CMT: 49; CMT: 69
Patient population	Adult (≥18 y), metastatic PD-L1 negative–NSCLC	Adult (≥18 y), metastatic or recurrent PD-L1 negative–NSCLC	Adult (≥18 y), metastatic or recurrent PD-L1 negative–NSCLC	Adult (≥18 y), metastatic nonsquamous PD-L1 negative–NSCLC	Adult (≥18 y), metastatic squamous PD-L1 negative–NSCLC	Adult (≥18 y), stage IIIB-IV nonsquamous PD-L1 negative–NSCLC
Country	Multinational	Multinational	Multinational	Multinational	Multinational	China
Regimen protocol	(1) DUR, TRE, and CMT Q3W × 4 cycles, then TRE on week 16 and DUR Q4W; (2) DUR and CMT Q3W × 4 cycles, then DUR Q4W; (3) CMT Q3W up to 6 cycles	(1) NIV Q3W and IPI Q6W and CMT Q3W × 2 cycles; (2) CMT Q3W × 4 cycles	(1) NIV Q3W and IPI Q6W; (2) NIV and CMT Q3W × 4 cycles; (3) CMT Q3W × 4 cycles	(1) PEM and CMT Q3W × 4 cycles, then PEM and pemetrexed maintenance; (2) PBO and CMT Q3W × 4 cycles, then PBO and pemetrexed maintenance	(1) PEM and CMT Q3W × 4 cycles, then PEM maintenance; (2) PBO and CMT Q3W × 4 cycles, then PBO maintenance	(1) CAM and CMT Q3W × 4-6 cycles, then CAM and pemetrexed maintenance; (2) CAM and CMT Q3W × 4-6 cycles, then pemetrexed maintenance
Survival end points	OS, PFS (dual primary)	OS (primary), PFS (secondary)	OS (primary), PFS (secondary)	OS, PFS (dual primary)	OS, PFS (dual primary)	PFS (primary), OS (secondary)
*EGFR*/*ALK* alteration	Excluded	Excluded	Excluded	Excluded	NA	Excluded
Histological subtype	Both nonsquamous and squamous	Both nonsquamous and squamous	Both nonsquamous and squamous	Nonsquamous	Squamous	Nonsquamous
Jadad score^a^	R: 1, D: 0, W: 1	R: 1, D: 0, W: 1	R: 1, D: 0, W: 1	R: 1, D: 1, W: 1	R: 1, D: 1, W: 1	R: 1, D: 0, W: 1

Abbreviations: *ALK*, anaplastic lymphoma kinase; CAM, camrelizumab; CMT, chemotherapy; D, double-blinding; DUR, durvalumab; *EGFR*, epidermal growth factor receptor; IPI, ipilimumab; NA, not available; NCT, ClinicalTrials.gov registry; NIV, nivolumab; NSCLC, non–small cell lung cancer; OS, overall survival; PBO, placebo; PD-L1, programmed death-ligand 1; PEM, pembrolizumab; PFS, progression-free survival; Q3W, every 3 weeks; Q4W, every 4 weeks; Q6W, every 6 weeks; R, randomization; TRE, tremelimumab; W, withdrawals and dropout.

^a^ Jadad score assesses the quality of randomized clinical trials; a higher score indicates higher quality.

**Figure.**
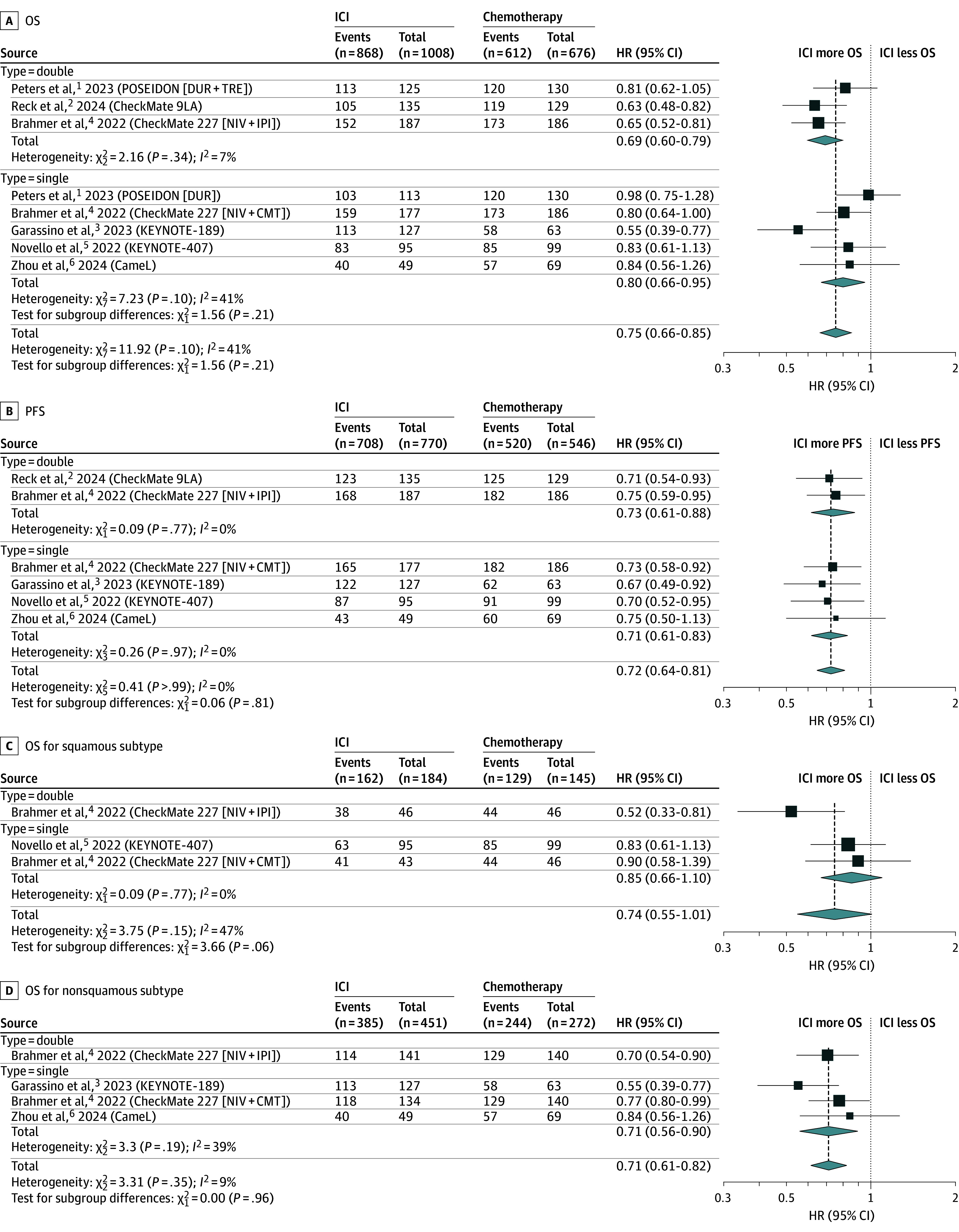
Immune Checkpoint Inhibitor (ICI)–Containing Regimen vs Chemotherapy (CMT) Alone in Metastatic Programmed Death-Ligand 1–Negative Non–Small Cell Lung Cancer (NSCLC) A, Five-year overall survival (OS); B, 5-year progression-free survival (PFS); C, 5-year OS for squamous histological subtype; and D, 5-year OS for nonsquamous histological subtype. CMT indicates chemotherapy; DUR, durvalumab; IPI, ipilimumab; NIV, nivolumab.

PFS was not different between the doublet ICI and the single ICI subgroup (pooled HR, 0.73; 95% CI, 0.61-0.88 vs HR, 0.71; 95% CI, 0.61-0.83; *P* for subgroup differences = .81). Egger tests did not show the presence of publication bias. The Grading of Recommendations, Assessment, Development, and Evaluations assessment showed moderate certainty of evidence.

Stratifying by histological subtype, we analyzed 3 RCTs on patients with squamous cell lung cancer. We found that doublet ICI (pooled HR, 0.52; 95% CI, 0.33-0.81), but not singlet ICI plus chemotherapy (pooled HR, 0.85; 95% CI, 0.66-1.10), was associated with improved OS.^3,5^ In contrast, patients with nonsquamous histological subtypes attained similar OS benefits from either doublet or singlet ICI (Figure, C and D).^3,4,6^

## Discussion

There is limited data on the outcomes of patients with negative PD-L1 expression and, to our knowledge, this is the first meta-analysis to address this question. Our analysis showed that the OS benefit from KEYNOTE-189 in patients with PD-L1–negative NSCLC was an outlier compared with similar studies. This is possibly due to differences in patient characteristics, such as inclusion of only nonsquamous histology, more patients with brain metastasis, and having less patient crossover to the ICI group upon disease progression. Other limitations of this meta-analysis included the small number of studies and the heterogeneity of the treatment regimen, particularly the difference in the number of chemotherapy cycles and the use of pemetrexed maintenance therapy in patients with nonsquamous histology. Also, doublet ICI regimen had better OS outcomes in patients with PD-L1–negative squamous cell lung cancer. Our findings suggest that doublet ICI regimens may be beneficial in patients with PD-L1–negative squamous cancer, while singlet ICI regimens may be preferred in nonsquamous histology. Further RCTs are needed to confirm the potential OS benefit of doublet ICI in the squamous histology subpopulation.

## Notes

Supplement 1. **eMethods**. **eAppendix.** List of search terms **eFigure. **PRISMA 2020 flow diagram
eReferences.

Supplement 2.
Data Sharing Statement
