# Supplementary material for: Long-Term Survival by Number of Immune Checkpoint Inhibitors in PD-L1–Negative Metastatic NSCLC: A Systematic Review and Meta-Analysis
Source: JAMA Netw Open. 2025 Feb 12;8(2):e2457357. doi: 10.1001/jamanetworkopen.2024.57357 (PMC11822538; doi:10.1001/jamanetworkopen.2024.57357)
Supplement: Supplement 1. — eMethods. eAppendix. List of search terms eFigure. PRISMA 2020 flow diagram eReferences. [file jamanetwopen-e2457357-s001.pdf]

Supplemental Online Content

Ponvilawan B, Bansal D, Abdel Karim N, Ramakrishnan R, Subramanian J. Long-term survival by number of immune checkpoint inhibitors in PD-L1–negative metastatic NSCLC: a systematic meta-analysis. *JAMA Netw Open*. 2025;8(2):e2457357. doi:10.1001/jamanetworkopen.2024.57357

eMethods.

eAppendix. List of search terms

eFigure. PRISMA 2020 flow diagram

eReferences

This supplemental material has been provided by the authors to give readers additional information about their work.

© 2025 Ponvilawan B et al. *JAMA Network Open*.

eMethods.

Data Sources and Searches

All available studies in the EMBASE, Ovid Medline, and Cochrane Central Register of Controlled Trials (CENTRAL) databases were retrieved from inception to July 2024. Search terms related to “metastatic NSCLC”, “ICI”, and “survival outcomes” were applied to select relevant studies (eTable 3). The study was registered in the INPLASY database (INPLASY202480120).

Selection Criteria

Eligible studies for this study must be phase III randomized control trials (RCTs) of patients with metastatic or recurrent NSCLC with negative PD-L1 status who did not receive any previous systemic therapy. At least one group from the study must receive ICI with or without chemotherapy as their first-line treatment, while one group received chemotherapy alone with or without placebo. Patients of both nonsquamous and squamous histological subtypes are eligible. For those with nonsquamous histological subtype, they must not have *EGFR* mutations or *ALK* rearrangements. They must report at least one of the outcomes of interest: overall survival (OS) and progression-free survival (PFS), as hazard ratios (HR) and their 95% confidence intervals (CI).

The eligibility of each study was independently reviewed by two investigators (B.P., N.A.K.). If decision conflicts occurred, the senior investigator (J.S.) determined the final decision. The Jadad quality assessment scoring system for randomized controlled studies was used to determine the quality of the included studies[1]. The Grading of Recommendations, Assessment, Development, and Evaluations (GRADE) approach was used to determine the certainty of evidence.

© 2025 Ponvilawan B et al. *JAMA Network Open*.

Definitions of Survival Outcomes

OS was defined as the time from randomization to death. PFS was defined as the time from randomization to disease progression or death.

Data Extraction

A standardized form containing the following information was utilized for data collection: the name of the RCT, NCT registration number, the last name of the first author, the publication year, the country of the study, the number of patients and treatment regimen in each arm, the survival endpoints of the study, the exclusion of patients with *EGFR* or *ALK* alterations, and the histological subtype (nonsquamous and squamous).

Statistical Analysis

We perform all statistical analyses using R version 4.3.2 software (Vienna, Austria) and "meta" version 7.0-0. Pooled hazard ratios (HR) and their 95% confidence intervals (CI) were calculated via the generic inverse variance method. Due to the high likelihood of interstudy heterogeneity, a random effects model was implemented for this meta-analysis. Statistical heterogeneity was determined using Cochran's Q test and I^2 statistic[2]. The presence of publication bias was determined with Egger's test. Sensitivity analysis of studies with singlet ICI was performed using the leave-one-out method.

© 2025 Ponvilawan B et al. *JAMA Network Open*.

eAppendix. List of search terms

EMBASE

1. 'non small cell lung cancer'/exp OR 'non small cell lung cancer'
2. nsclc
3. 'squamous cell lung carcinoma'/exp OR 'squamous cell lung carcinoma'
4. 'lung adenocarcinoma'/exp OR 'lung adenocarcinoma'
5. 'large cell lung carcinoma'/exp OR 'large cell lung carcinoma'
6. 'lung adenosquamous carcinoma'/exp OR 'lung adenosquamous carcinoma'
7. #1 OR #2 OR #3 OR #4 OR #5 OR #6
8. 'immune checkpoint inhibitor'/exp OR 'immune checkpoint inhibitor'
9. 'pd1 antibody'/exp OR 'pd1 antibody'
10. 'pd l1 antibody'/exp OR 'pd l1 antibody'
11. 'cytotoxic t lymphocyte antigen 4 antibody'/exp OR 'cytotoxic t lymphocyte antigen 4 antibody'
12. 'pembrolizumab'/exp OR 'pembrolizumab' OR 'nivolumab'/exp OR 'nivolumab' OR 'cemiplimab'/exp OR 'cemiplimab' OR 'atezolizumab'/exp OR 'atezolizumab' OR 'durvalumab'/exp OR 'durvalumab' OR 'ipilimumab'/exp OR 'ipilimumab' OR 'ticilimumab'/exp OR 'ticilimumab' OR 'ipilimumab plus nivolumab'/exp OR 'ipilimumab plus nivolumab' OR 'mk 3475'/exp OR 'mk 3475' OR 'keytruda'/exp OR Keytruda OR 'opdivo'/exp OR opdivo OR 'ono 4538'/exp OR 'ono 4538' OR 'bms 936558'/exp OR 'bms 936558' OR 'mdx1106'/exp OR mdx1106 OR 'regn 2810'/exp OR 'regn 2810' OR 'libtayo'/exp OR libtayo OR 'rg7446'/exp OR rg7446 OR 'mpdl3280a'/exp OR mpdl3280a OR 'tecentriq'/exp OR tecentriq OR 'medi4736'/exp OR medi4736 OR 'imfinzi'/exp OR Imfinzi OR 'bms 734016'/exp OR 'bms 734016' OR 'mdx 010'/exp OR 'mdx 010' OR 'mdx 101'/exp OR 'mdx 101' OR 'yervoy'/exp OR yervoy OR 'cp 675206'/exp OR 'cp 675206' OR imjudo OR ‘camrelizumab’/exp OR ‘camrelizumab’ OR ‘shr 1210’/exp OR ‘shr 1210’OR ‘airuika’/exp OR ‘airuika’ OR ‘toripalimab’/exp OR ‘toripalimab’OR ‘bgb a 317’/exp OR ‘bgb a 317’ OR ‘sintilimab’/exp OR ‘sintilimab’ OR ‘tyvyt’/exp OR tyvyt OR ‘ibi308’/exp OR ibi308
13. #8 OR #9 OR #10 OR #11 OR #12
14. 'randomized controlled trial'/de OR 'controlled clinical trial'/de OR random*:ti,ab,tt OR 'randomization'/de OR 'intermethod comparison'/de OR placebo:ti,ab,tt OR compare:ti,tt OR compared:ti,tt OR comparison:ti,tt OR (open NEXT/1 label):ti,ab,tt OR 'double blind procedure'/de OR (parallel NEXT/1 group*):ti,ab,tt OR crossover:ti,ab,tt OR 'cross over':ti,ab,tt OR ((assign* OR match OR matched OR allocation) NEAR/6 (alternate OR group OR groups OR intervention OR interventions OR patient OR patients OR subject OR subjects OR participant OR participants)):ti,ab,tt OR ((double OR single OR doubly OR singly) NEXT/1 (blind OR blinded OR blindly)):ti,ab,tt OR assigned:ti,ab,tt OR allocated:ti,ab,tt OR (controlled NEAR/8 (study OR design OR trial)):ti,ab,tt OR volunteer:ti,ab,tt OR volunteers:ti,ab,tt OR 'human experiment'/de OR trial:ti,tt OR ((evaluated:ab OR evaluate:ab OR evaluating:ab OR

© 2025 Ponvilawan B et al. *JAMA Network Open*.

assessed:ab OR assess:ab) AND (compare:ab OR compared:ab OR comparing:ab OR comparison:ab))

15. ((random* NEXT/1 sampl* NEAR/8 ('cross section*' OR questionnaire* OR survey OR surveys OR database OR databases)):ti,ab,tt) NOT ('comparative study'/de OR 'controlled study'/de OR 'randomised controlled':ti,ab,tt OR 'randomized controlled':ti,ab,tt OR 'randomly assigned':ti,ab,tt) OR ('cross-sectional study' NOT ('randomized controlled trial'/de OR 'controlled clinical study'/de OR 'controlled study'/de OR 'randomised controlled':ti,ab,tt OR 'randomized controlled':ti,ab,tt OR 'control group':ti,ab,tt OR 'control groups':ti,ab,tt)) OR ('case control*':ti,ab,tt AND random*:ti,ab,tt NOT ('randomised controlled':ti,ab,tt OR 'randomized controlled':ti,ab,tt) OR 'update review':ab OR (databases NEAR/5 searched):ab) OR ('systematic review':ti,tt NOT (trial:ti,tt OR study:ti,tt)) OR (nonrandom*:ti,ab,tt NOT random*:ti,ab,tt) OR 'random field*':ti,ab,tt OR ('random cluster' NEAR/4 sampl*):ti,ab,tt OR (review:ab AND review:it NOT trial:ti,tt) OR ('we searched':ab AND (review:ti,tt OR review:it)) OR ((rat:ti,tt OR rats:ti,tt OR mouse:ti,tt OR mice:ti,tt OR swine:ti,tt OR porcine:ti,tt OR murine:ti,tt OR sheep:ti,tt OR lambs:ti,tt OR pigs:ti,tt OR piglets:ti,tt OR rabbit:ti,tt OR rabbits:ti,tt OR cat:ti,tt OR cats:ti,tt OR dog:ti,tt OR dogs:ti,tt OR cattle:ti,tt OR bovine:ti,tt OR monkey:ti,tt OR monkeys:ti,tt OR trout:ti,tt OR marmoset*:ti,tt) AND 'animal experiment'/de) OR ('animal experiment'/de NOT ('human experiment'/de OR 'human'/de))
16. #14 NOT #15
17. ‘metastasis’/exp OR ‘metastasis’
18. ‘advanced cancer’/exp OR ‘advanced cancer’
19. ‘cancer recurrence’/exp OR ‘cancer recurrence’
20. #17 OR #18 OR #19
21. #7 AND #13 AND #16 AND #20

Ovid Medline

1. non small cell lung cancer.mp. or exp Carcinoma, Non-Small-Cell Lung/
2. lung adenocarcinoma.mp. or exp "Adenocarcinoma of Lung"/
3. exp Carcinoma, Squamous Cell/ or squamous cell carcinoma lung.mp.
4. large cell carcinoma lung.mp. or exp Carcinoma, Large Cell/
5. exp Carcinoma, Adenosquamous/ or adenosquamous carcinoma lung.mp.
6. nsclc.mp.
7. immune checkpoint inhibitor.mp. or exp Immune Checkpoint Inhibitors/
8. exp Programmed Cell Death 1 Receptor/ or pd1 antibody.mp.
9. exp B7-H1 Antigen/ or pdl1 antibody.mp.
10. exp CTLA-4 Antigen/ or ctla4 antibody.mp.
11. nivolumab.mp. or exp Nivolumab/
12. ipilimumab.mp. or exp Ipilimumab/
13. pembrolizumab.mp. or cemiplimab.mp. or atezolizumab.mp. or durvalumab.mp. or tremelimumab.mp. or ticilimumab.mp. or mk-3475.mp. or keytruda.mp. or opdivo.mp. or ono-4538.mp. or bms-936558.mp. or mdx1106.mp. or regn2810.mp. or libtayo.mp. or rg7446.mp. or

© 2025 Ponvilawan B et al. *JAMA Network Open*.

mpdl3280a.mp. or tecentriq.mp. or medi4736.mp. or imfinzi.mp. or bms-734016.mp. or mdx-010.mp. or mdx-101.mp. or yervoy.mp. or cp-675206.mp. or imjudo.mp. or camrelizumab.mp. or SHR-1210.mp. or airuika.mp. or toripalimab.mp. or tuoyi.mp. or js001.mp. or tislelizumab.mp. or BGB-A317.mp. or sintilimab.mp. or tyvyt.mp. or ibi308.mp.
14. randomized controlled trial.pt. or controlled clinical trial.pt. or randomized.ab. or placebo.ab. or drug therapy.fs. or randomly.ab. or trial.ab. or groups.ab.
15. exp animals/ not humans.sh.
16. 14 not 15
17. metastasis.mp. or exp Neoplasm Metastasis/
18. advanced cancer.mp. or cancer recurrence.mp.
19. 1 or 2 or 3 or 4 or 5 or 6
20. 11 or 12 or 13
21. 17 or 18
22. 16 and 19 and 20 and 21

CENTRAL

1. MeSH descriptor: [Carcinoma, Non-Small-Cell Lung] explode all trees
2. MeSH descriptor: [Adenocarcinoma of Lung] explode all trees
3. MeSH descriptor: [Carcinoma, Large Cell] explode all trees
4. MeSH descriptor: [Carcinoma, Adenosquamous] explode all trees
5. MeSH descriptor: [Carcinoma, Squamous Cell] explode all trees
6. MeSH descriptor: [Immune Checkpoint Inhibitors] explode all trees
7. MeSH descriptor: [Programmed Cell Death 1 Receptor] explode all trees
8. MeSH descriptor: [B7-H1 Antigen] explode all trees
9. MeSH descriptor: [CTLA-4 Antigen] explode all trees
10. MeSH descriptor: [Nivolumab] explode all trees
11. MeSH descriptor: [Ipilimumab] explode all trees
12. 'pembrolizumab' or 'cemiplimab' or 'atezolizumab' or 'durvalumab' or ' tremelimumab' or 'ticilimumab' or 'camrelizumab' or 'toripalimab' or 'tislelizumab' or 'sintilimab' or 'mk-3475' or 'keytruda' or 'opdivo' or 'ono-4538' or 'bms-936558' or 'mdx1106' or 'regn2810' or 'libtayo' or 'rg7446' or 'mpdl3280a' or 'tecentriq' or 'medi4736' or 'imfinzi' or 'bms-734016' or 'mdx-010' or 'mdx-101' or 'yervoy' or 'cp-675206' or 'imjudo' or 'SHR-1210' or 'airuika' or 'tuoyi' or 'js001' or 'BGB-A317' or 'tyvyt' or 'ibi308'
13. MeSH descriptor: [Neoplasm Metastasis] explode all trees
14. 'advanced cancer' or 'cancer recurrence'
15. MeSH descriptor: [Randomized Controlled Trial] explode all trees
16. MeSH descriptor: [Clinical Trials, Phase III as Topic] explode all trees
17. MeSH descriptor: [Randomized Controlled Trials as Topic] explode all trees
18. #1 or #2 or #3 or #4 or #5 or #6
19. #7 or #8 or #9 or #10 or #11 or #12
20. #13 or #14

© 2025 Ponvilawan B et al. *JAMA Network Open*.

21. #15 or #16 or #17
22. #18 and #19 and #20 and #21

© 2025 Ponvilawan B et al. *JAMA Network Open*.

eFigure. PRISMA 2020 flow diagram

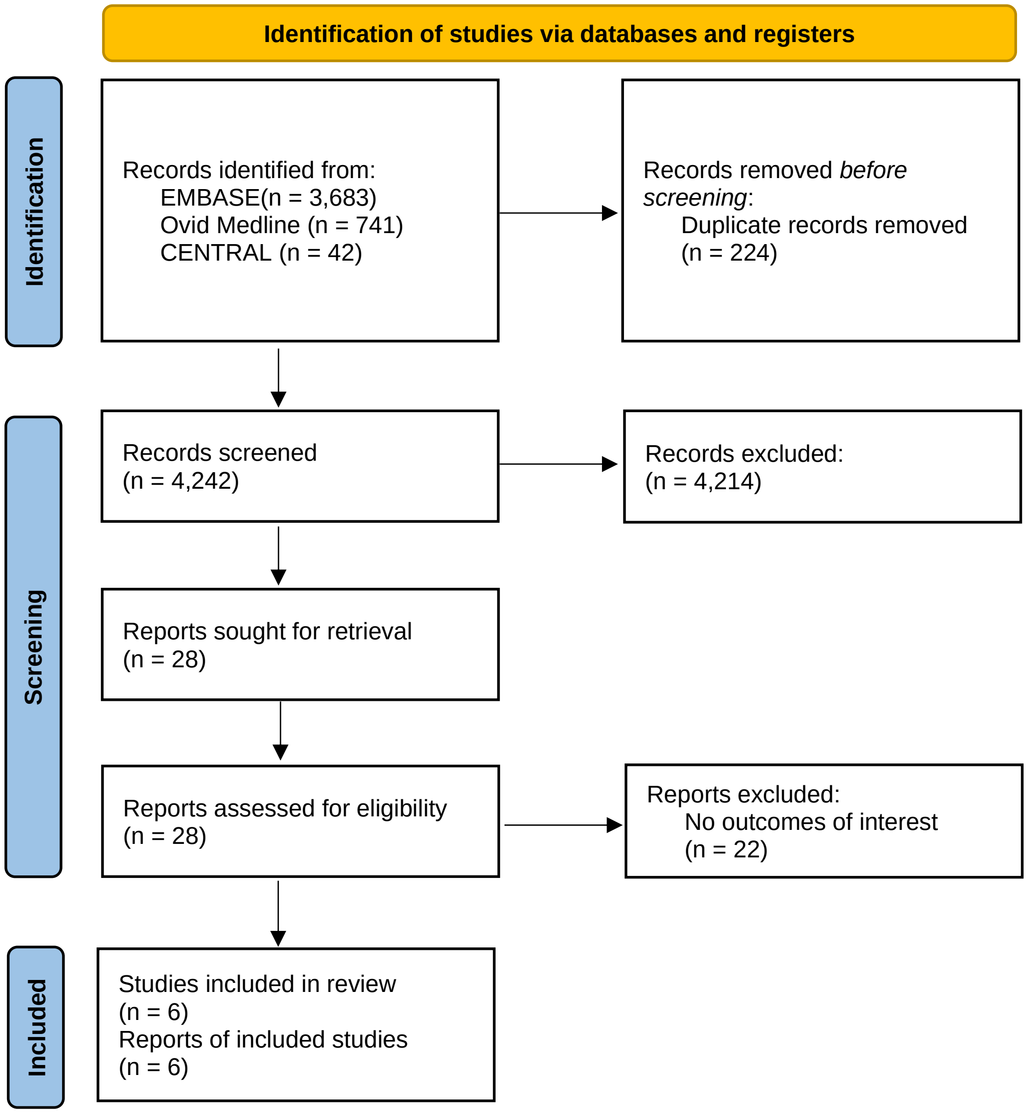

Source: Page MJ, et al. BMJ 2021;372:n71. doi: 10.1136/bmj.n71.

This work is licensed under CC BY 4.0. To view a copy of this license, visit https://creativecommons.org/licenses/by/4.0/

eReferences

1. Jadad AR, Moore RA, Carroll D, et al. Assessing the quality of reports of randomized clinical trials: Is blinding necessary? *Controlled Clinical Trials*. 1996/02/01/ 1996;17(1):1-12. doi:https://doi.org/10.1016/0197-2456(95)00134-4
2. Higgins JP, Thompson SG, Deeks JJ, Altman DG. Measuring inconsistency in meta-analyses. *Bmj*. Sep 6 2003;327(7414):557-60. doi:10.1136/bmj.327.7414.557

© 2025 Ponvilawan B et al. *JAMA Network Open*.
