# Supplementary material for: Long-Term Survival by Number of Immune Checkpoint Inhibitors in PD-L1–Negative Metastatic NSCLC: A Systematic Review and Meta-Analysis
Source: JAMA Netw Open. 2025 Feb 12;8(2):e2457357. doi: 10.1001/jamanetworkopen.2024.57357 (PMC11822538; doi:10.1001/jamanetworkopen.2024.57357)
Supplement: Supplement 2. — Data Sharing Statement [file jamanetwopen-e2457357-s002.pdf]

Data Sharing Statement

Ponvilawan. Long-Term Survival by Number of Immune Checkpoint Inhibitors in PD-L1–Negative Metastatic NSCLC. *JAMA Netw Open*. Published February 06, 2025. doi:10.1001/jamanetworkopen.2024.57357

Data
Data available: No

Additional Information
Explanation for why data not available: Data is publicly available.
